# Effects of Dietary Glutamine Supplementation on the Body Composition and Protein Status of Early-Weaned Mice Inoculated with *Mycobacterium bovis* Bacillus Calmette-Guerin

**DOI:** 10.3390/nu3090792

**Published:** 2011-08-31

**Authors:** Marcelo Macedo Rogero, Maria Carolina Borges, Inar Alves de Castro, Ivanir S. O. Pires, Primavera Borelli, Julio Tirapegui

**Affiliations:** 1 Department of Nutrition, School of Public Health, University of Sao Paulo, Sao Paulo 01246-904, Brazil; Email: mcarolborges@usp.br; 2 Department of Food and Experimental Nutrition, Faculty of Pharmaceutical Sciences, University of Sao Paulo, Sao Paulo 05508-900, Brazil; Email: inar@usp.br (I.A.C.); ivanirpires@yahoo.com.br (I.S.O.P.); tirapegu@usp.br (J.T.); 3 Department of Clinical and Toxicological Analyses, Faculty of Pharmaceutical Sciences, University of Sao Paulo, Sao Paulo 05508-900, Brazil; Email: borelli@usp.br

**Keywords:** glutamine, early weaning, body composition, mice

## Abstract

Glutamine, one of the most abundant amino acids found in maternal milk, favors protein anabolism. Early-weaned babies are deprived of this source of glutamine, in a period during which endogenous biosynthesis may be insufficient for tissue needs in states of metabolic stress, mainly during infections. The objective of this study was to verify the effects of dietary glutamine supplementation on the body composition and visceral protein status of early-weaned mice inoculated with *Mycobacterium bovis* Bacillus Calmette-Guérin (BCG). Mice were weaned early on their 14th day of life and seperated into two groups, one of which was fed a glutamine-free diet (*n* = 16) and the other a glutamine-supplemented diet (40 g/kg diet) (*n* = 16). At 21 days of age, some mice were intraperitoneally injected with BCG. Euthanasia was performed at the 28th day of age. BCG inoculation significantly reduced body weight (*P* < 0.001), lean mass (*P* = 0.002), water (*P* = 0.006), protein (*P* = 0.007) and lipid content (*P* = 0.001) in the carcass. Dietary glutamine supplementation resulted in a significant increase in serum IGF-1 (*P* = 0.019) and albumin (*P* = 0.025) concentration, muscle protein concentration (*P* = 0.035) and lipid content (*P* = 0.002) in the carcass. In conclusion, dietary glutamine supplementation had a positive influence on visceral protein status but did not affect body composition in early-weaned mice inoculated with BCG.

## 1. Introduction

Weaning (the period in which infants transition from a diet of milk to other food) is when infants are at the highest risk of malnutrition, infections, and growth faltering [1]. Glutamine and glutamate are the most abundant amino acids found in proteins and in human milk, as well as in rat and mouse milk [2,3]. Furthermore, as human lactation progresses, glutamine and glutamate increase approximately 20 and 2.5 times, respectively, representing more than 50% of all free amino acids in human milk by the third month of lactation [4]. On average only about 35% of infants 0 to 6 months old are exclusively breastfed [5]. Exclusive breastfeeding is rarely sustained, and often supplemented with other formula or other complementary foods. In contrast to infants fed with maternal milk, premature or early-weaned infants are deprived of this glutamine source, which renders them exclusively dependent on endogenous synthesis of this amino acid or on supplementation by means of commercially available formulations for babies [4].

The high concentration of glutamine in maternal milk may be related to the role of this amino acid in the metabolism of rapidly dividing cells such as enterocytes and leucocytes [6]. Within this context, Rogero *et al.* [7,8,9] showed that a lack of glutamine intake associated with early weaning hinders the functionality of peritoneal macrophages, an effect which could be partially reversed by glutamine ingestion. Furthermore, glutamine supplementation reduces the incidence of infectious morbidity in preterm and low-birth-weight neonates [10,11].

Glutamine is considered a conditionally essential amino acid in catabolic stress states [12], when muscle stores of glutamine become depleted [13]. There is evidence that glutamine supplementation may influence nitrogen balance and prognosis in some critical patients [14,15]. In addition, dietary glutamine supplementation in rodents reversed the reduction in muscle and liver protein and DNA concentration induced by early weaning [8]. Since the effect of glutamine supplementation on protein balance and body composition of early-weaned animals under situations of metabolic stress is unknown, the present study aimed to investigate the effects of dietary glutamine supplementation on the body composition and visceral protein status, as defined by serum albumin and IGF-1 levels, of early-weaned mice inoculated with *Mycobacterium bovis* Bacillus Calmette-Guérin (BCG), a weakened strain of *Mycobacterium bovis* used as a vaccine against tuberculosis, which promotes a inflammatory response [16], as well as an increase in body protein catabolism [17] in rodents. To date, studies relating BCG and glutamine have evaluated only the effect of glutamine supplementation *in vitro* on the function of peritoneal macrophages activated with BCG, as well as on the pattern of cytokine production from peripheral blood mononuclear cells [18,19,20].

## 2. Experimental Section

Mice and treatments. Male Swiss Webster mice obtained from the Animal Laboratory of the Faculty of Pharmaceutical Sciences at the University of São Paulo were weaned at 14 day of age. The mice were kept in a room at an ambient temperature of 22 ± 2 °C and a relative humidity of 55 ± 10% under a 12-h light/12-h dark cycle (lights on at 0700). The mice were weighed daily and their final mass was recorded immediately before sacrifice.

All mice were generated by mating two 2-month-old primiparous females with a single male. After pregnancy was confirmed, the females (*n* = 8) were maintained isolated in individual cages throughout gestation. After the birth of their offspring, eight males were chosen and then maintained with their mother during the next 14 day. At the time of weaning (at the age of 14 day), 32 baby mice, 4 from each female, were distributed into two groups: 16 of them were fed a glutamine-free diet (−Gln), while the other 16 were fed a glutamine-supplemented diet (+Gln). Mice of the −Gln (*n* = 16) and +Gln (*n* = 16) groups were fed ad libitum with water and a diet made specifically for infant mice, according to the American Institute of Nutrition (AIN-93G) [21] from day 14 to day 28 (Table 1). It is important to emphasize that 14 day old mice are able to process solid food. In order to ensure that the diet was glutamine-free, a casein substitute was formulated using a mixture of amino acids without glutamine (Ajinomoto Interamericana Indústria e Comércio Ltda, São Paulo, Brazil) in similar quantities to those found in casein (Table 2). The amount of nitrogen corresponding to the withdrawal of glutamine (3.33 g nitrogen/kg diet) was substituted with the dispensable amino acids alanine, aspartic acid, glycine, proline and serine. Mice from the +Gln group were also fed a casein-free diet, which was supplemented by the addition of each one of the amino acids present in casein plus additional glutamine (40 g glutamine/kg diet). This dose of glutamine was chosen based on some studies [22,23,24]. 

**Table 1:** Diet compositions ^1,2^.

Ingredient	−Gln diet	+Gln diet
	g/kg diet	
Cornstarch	542.4	562.1
Amino acid mixture	190.1	170.4
Sucrose	100	100
Soybean oil	70	70
Fiber source (cellulose)	50	50
Mineral mixture ^3,4^	35	35
Vitamin mixture ^5^	10	10
Choline bitartrate (41.1% choline)	2.5	2.5
*tert*-Butylhydroquinone	0.014	0.014

^1^ Based on AIN-93G [21]; ^2^ Both diets (−Gln and +Gln) are isocaloric (1674 kJ/100 g) and isonitrogenous (2.45 g nitrogen/100 g); ^3^ Due to the removal of casein from the formulation of the diet, the mineral mixture was enriched with a 180.68 g potassium phosphate/kg mix, resulting in the supply of 3 g phosphorus/kg diet; ^4^ AIN-93G [21] mineral mixture; ^5^ AIN-93G [21] vitamin mixture.

**Table 2:** Amino acid profiles of the diets ^1^.

Amino acid	−Gln diet	+Gln diet
	g/kg diet	
Nonessential		
L-Ala	8.8	2.6
L-Asp	18.5	3.9
L-Glu	18.9	18.9
Gly	6.8	2.2
L-Gln	-	40.0
L-Pro	26.0	3.4
L-Ser	14.7	3.1
NEAA-N ^2^	11.52	11.52
Essential		
L-Arg	6.4	6.4
L-Cys	3.7	3.7
L-Phe	8.8	8.8
L-His	4.6	4.6
L-Ile	8.5	8.5
L-Leu	15.4	15.4
L-Lys	16.2	16.2
L-Met	4.6	4.6
L-Thr	6.7	6.7
L-Tyr	9.3	9.3
L-Trp	2.1	2.1
L-Val	10.0	10.0
EAA-N ^2^	12.94	12.94
NEAA-N:EAA-N ratio, g:g	1.12	1.12

^1^ Based on the composition of amino acids present in the AIN-93G diet [21]; ^2^ NEAA-N, nonessential amino acid nitrogen; EAA-N, essential amino acid nitrogen.

At 21 day of age, eight mice from the −Gln group and eight mice from the +Gln group were intraperitoneally inoculated with 10^7^ viable units of BCG, *Mycobacterium bovis* cepa Moreau, provided by the Butantan Institute (Sao Paulo, SP, Brazil). Thus, the study was performed with four experimental groups: −Gln/−BCG (*n* = 8), −Gln/+BCG (*n* = 8), +Gln/−BCG (*n* = 8) and +Gln/+BCG (*n* = 8).

On day 28, the mice from the four groups were intraperitoneally anesthetized with ketamine hydrochloride (100 mg/kg body mass) in combination with xylazine hydrochloride (50 mg/kg body mass) and then killed by the cervical dislocation method. All of the mice were sacrificed in the morning between 0800 and 1200. Blood, spleen, liver, and muscle tissue from the gastrocnemius were collected for analyses. All procedures carried out on mice were approved by the Ethics Committee on Animal Experimentation of the Faculty of Pharmaceutical Sciences, University of Sao Paulo (3 June 2003; protocol number 14), according to the guidelines on Animal Experimentation of the Brazilian College. 

### 2.1. Serum Determinations

Serum albumin concentration was assayed by the bromocresol green method [25]. Serum protein and iron concentration was measured according to the methods of Biuret [26] and Goodwin [27], respectively, using commercial kits (CELM, São Paulo, Brazil; Labtest Diagnostica SA, São Paulo, Brazil). Serum corticosterone and IGF-1 concentration was measured with a commercially available radioimmunoassay kit (DSL, Diagnostic Systems Laboratories, Inc., Webster, TX, USA). The radioactivity was determined in a gamma counter (Beckman L600). The mice used for the determination of the serum concentration of corticosterone were sacrificed using a guillotine, in order to minimize any alteration in the serum concentration of corticosterone induced by the use of anesthetics. The plasma glutamine concentration was determined according to the method described by Lund [28], based on the enzymatic conversion of NAD^+^ to NADH, which is directly proportional to the initial glutamine content. 

### 2.2. Tissue Determinations

Muscle glutamine was extracted as described by Sahlin *et al.* [29]; frozen samples were pulverized, homogenized in cold perchloric acid (PCA) (0.5 M) and centrifuged (3000 rpm, 4 °C, 15 min). Supernatant was neutralized with sodium bicarbonate (0.5 M) and used for glutamine determination as described by Lund [28]. The maximal activity of the enzyme glutamine synthetase (GS) in the liver was determined according to Minet *et al.* [30]. Briefly, liver was homogenized in a solution containing 50 mM of imidazole. After centrifugation, the supernatant was used for the enzymatic assay. The assay mixture consisted of 50 mM imidazole (pH 6.8), 50 mM L-glutamine, 25 mM hydroxylamine, 25 mM sodium arsenate, 2 mM manganese chloride and 0.16 mM diphosphate adenosine. After 30 min incubation at 37 °C, reaction was blocked, samples centrifuged and absorbance was measured at 540 nM. For the extraction of protein and RNA, tissue samples were pulverized, homogenized in trichloroacetic acid 10%, and centrifuged (3000 rpm, 4 °C, 15 min). The pellet was resuspended in cold PCA 2% and centrifuged (3000 rpm, 4 °C, 15 min). The resulting pellet was resuspended in sodium hydroxide (0.3 N) and incubated at 37 °C for 1 h. After this period, an aliquote of this solution was used for the determination of protein according to the method described by Lowry *et al.* [31]. Cold PCA 20% was added to the remaining solution, which was incubated on ice for 10 min, and, then, centrifuged (3000 rpm, 4 °C, 15 min). The supernatant was used for the determination of RNA in the tissues according to the method described by Munro and Fleck [32]. 

### 2.3. Body Composition Determination

The body composition of the animals (fat, protein, humidity, and ashes) was determined by chemical analysis of the carcass. Humidity content was determined by drying the whole carcass in a ventilated oven (70 °C) for 7 day. Carcasses were weighed before being placed in the oven and after being dried, and the difference between measurements was considered to be the absolute humidity content. The whole dry carcass was then chopped up and wrapped in gauze and filter paper for determination of body fat by the solvent extraction technique using a Soxhlet apparatus and ethyl ether as solvent. The remaining carcass without humidity and fat was completely ground (IKA M20 grinder, Labortechnik, Wasserburg, Germany) and sieved for the removal of hair, which could decrease the homogeneity of the sample. This process resulted in a highly homogenous powder that was used to determine carcass protein by the micro-Kjeldahl method [33] and ash content. Two grams of powder was placed in a muffle furnace for 12 h at 550 °C and then cooled, and the ash weight of the sample was determined. The amount of lean mass was calculated by subtracting absolute fat mass from total carcass mass. Fat, protein, humidity, ash, and lean mass contents were also calculated in percentages in relation to carcass mass.

### 2.4. Statistical Analyses

Initial body weight was expressed as means (Pooled SD) and analyzed by one-way ANOVA after checking the homogeneity of variances using a Bartlett test. The Tukey HSD was applied to compare means. The effects of glutamine supplementation (diet) and inoculation with BCG were expressed as means followed by Pooled SD and analyzed by factorial ANOVA (2^2^) followed by a Tukey HSD test. Probability values of *P* < 0.05 indicated statistical significance. Calculations were performed using Statistica version 7.1 (StatSoft Inc., Tulsa, OK, USA).

## 3. Results

Mean daily diet intakes (3.2 ± 0.5 g) were not different among groups. Despite the fact that the initial body weight (8.2 ± 0.7 g) did not differ among groups, BCG inoculation significantly reduced (*P* < 0.001) final body weight (−BCG = 18.6 ± 0.4 g; +BCG = 16.0 ± 0.6 g). BCG inoculation also significantly decreased serum iron concentration (*P* < 0.001). The glutamine-supplemented diet resulted in an increase in serum IGF-1 (*P* = 0.019) and albumin (*P* = 0.025) concentration, serum IGF-1 concentration being higher in the +Gln/+BCG group compared to the −Gln/+BCG group (Table 3). Serum corticosterone and total protein concentration were not affected by either diet or BCG inoculation. The plasma glutamine concentration tended to be greater (*P* = 0.054) in the +Gln groups than in the −Gln groups but was not affected by BCG.

BCG inoculation had a marked effect on body composition, since it significantly decreased carcass mass (*P* < 0.001), lean mass (*P* = 0.002), and water (*P* = 0.006), protein (*P* = 0.007) and lipid (*P* = 0.001) content, while glutamine supplementation increased the lipid content of the carcass (*P* = 0.002) (Table 4). BCG inoculation led to a significant reduction in muscle weight (*P* = 0.041) and liver RNA concentration (*P* = 0.021) and a significant increase in spleen weight (*P* < 0.001) and the liver (*P* = 0.008) and muscle (*P* = 0.018) protein:RNA ratio. Glutamine supplementation increased liver weight (*P* = 0.032) and muscle protein concentration (*P* = 0.035). Muscle glutamine concentration and the maximal activity of GS in the liver were not affected by either diet or BCG inoculation (Table 5).

**Table 3:** Plasma/serum variables of 28-day-old rats weaned to glutamine-supplemented (+Gln) or glutamine-free (−Gln) diets at 14 days of age and intraperitoneally injected (+BCG) or not injected (−BCG) with BCG at 21 days of age. São Paulo, 2007.

	Experimental Groups							
Variable ^1^	−Gln −BCG	–Gln +BCG	+Gln −BCG	+Gln +BCG	Pooled SD	Diet ^2^	Infection ^2^	Diet × Infection ^2^
Serum albumin (g/dL)	3.26	3.23	3.44	3.39	0.04	0.025	0.537	0.892
Serum total protein (g/dL)	4.11	4.27	4.34	4.19	0.05	0.487	0.994	0.140
Plasma glutamine (μmol/L)	0.52	0.52	0.57	0.58	0.01	0.054	0.890	0.781
Serum iron (µg/dL)	231.0	155.3	210.4	152.1	8.5	0.413	<0.001	0.548
Serum IGF-1 (ng/mL)	277.5	173.8	274.7	333.7	18.5	0.019	0.480	0.016
Serum corticosterone (ng/mL)	92.5	94.9	112.3	96.5	6.2	0.659	0.387	0.299

^1^ Values are means and variation of the groups is expressed as pooled SD, *n* = 8; ^2^ Probability values obtained by factorial ANOVA.

**Table 4:** Mass and chemical composition of the carcasses of 28-day-old rats weaned to glutamine-supplemented (+Gln) or glutamine free (−Gln) diets at 14 days of age and intraperitoneally injected (+BCG) or not injected (−BCG) with BCG at 21 days of age. São Paulo, 2007.

	Experimental Groups								
Variable ^1^	−Gln −BCG	−Gln +BCG	+Gln –BCG	+Gln +BCG		Pooled SD	Diet ^2^	Infection ^2^	Diet × Infection ^2^
Carcass mass (g)	18.38	16.16	19.81	16.08		2.57	0.359	<0.001	0.302
Lean mass (g)	16.96	14.10	16.87	14.25		2.27	0.966	0.002	0.880
Humidity (g)	11.59	10.62	12.71	10.51		1.78	0.365	0.006	0.271
Protein (g)	3.34	2.93	3.34	2.77		0.55	0.619	0.007	0.644
Fat (g)	1.28	1.09	1.77	1.23		0.36	0.002	0.001	0.076
Ashes (g)	0.80	0.66	0.79	0.65		0.23	0.937	0.088	0.979

^1^ Values are means and variation of the groups is expressed as pooled SD, *n* = 8; ^2^ Probability values obtained by factorial ANOVA.

**Table 5:** Tissue weights, RNA, and protein concentration of 28-day-old rats weaned to glutamine-supplemented (+Gln) or glutamine free (−Gln) diets at 14 days of age and intraperitoneally injected with BCG (+BCG) or not (−BCG) at 21 days of age. São Paulo, 2007.

	Experimental Groups								
Variable ^1^	−Gln −BCG	−Gln +BCG	+Gln −BCG	+Gln +BCG	Pooled SD		Diet ^2^	Infection ^2^	Diet × Infection ^2^
Liver (g)	1.03	1.07	1.17	1.17	0.05		0.032	0.685	0.602
Gastrocnemius Muscle (g)	0.071	0.070	0.085	0.067	0.010		0.175	0.041	0.055
Spleen (g)	0.12	0.27	0.14	0.29	0.08		0.321	<0.001	0.894
Liver Protein (mg/100 mg tissue)	12.87	13.35	13.52	13.52	0.88		0.436	0.329	0.426
Muscle Protein (mg/100 mg tissue)	14.15	14.01	14.34	15.93	0.27		0.035	0.137	0.082
Spleen Protein (mg/100 mg tissue)	12.80	13.63	14.45	13.42	1.28		0.188	0.846	0.093
Liver Protein/RNA ratio	0.013	0.015	0.013	0.014	0.00		0.458	0.008	0.584
Muscle Protein/RNA ratio	0.051	0.060	0.057	0.063	0.01		0.336	0.018	0.804
Spleen Protein/RNA ratio	0.013	0.015	0.014	0.014	0.00		0.940	0.573	0.606
Liver RNA (µg/g fresh tissue)	1,023	913	1,076	977	107.88		0.186	0.021	0.977
Muscle RNA (µg/g fresh tissue)	278	242	254	254	31.89		0.750	0.144	0.177
Spleen RNA (µg/g fresh tissue)	991	940	1,080	984	105.20		0.179	0.136	0.373
Muscle glutamine (µmol glutamine/g fresh tissue)	3.91	3.94	4.16	3.76	0.12		0.904	0.453	0.389
Liver Glutamine synthetase (nmol/h/mg protein)	1,294	862	1,195	933	465		0.934	0.050	0.618

^1^ Values are means and variation of the groups is expressed as pooled SD, *n* = 8; ^2^ Probability values obtained by factorial ANOVA.

## 4. Discussion

Glutamine is a conditionally essential amino acid because of the body’s inability to synthesize sufficient amounts to keep up with the demands during acute stress situation [34,35]. In the present study, we investigated the effect of dietary glutamine supplementation on the body composition and visceral protein status of early-weaned mice inoculated with BCG. Glutamine supplementation in rodents is usually done through diet or by intragastric administration. Nevertheless, in this study, intragastric administration would result in serious problems of administration of glutamine, due to the small size of the animals. At the same time, several published studies show that glutamine supplementation through the diet is effective in promoting beneficial effect on the immune response and reduction of mortality rate [22,23,24].

Dietary glutamine supplementation for 14 days significantly improved visceral protein status in early-weaned mice inoculated with BCG. Within this context, glutamine supplementation increased serum IGF-1 and albumin concentration. Serum IGF-1 concentration is a widely used biomarker for nutritional status assessment; since it is sensitive to diet composition and protein content [36]. Low protein intake may decrease serum IGF-1 concentration, which seems to be related to reductions in liver IGF-1 mRNA content [37]. Additionally, Takenaka *et al.* [38] showed that dietary restriction of single essential amino acids reduces serum IGF-1 concentration. In the present study, diets were isonitrogenous and had the same essential-to-nonessential amino acid ratio. Since there were no differences in diet consumption among groups, it is suggested that glutamine might be involved in the modulation of IGF-1 metabolism in early-weaned animals under metabolic stress.

IGF-1 promotes muscle protein synthesis, which contributes to a positive protein balance [39]. Accordingly, the higher muscle protein concentration observed in the +GLN/+BCG group compared to −GLN/+BCG group might be related to the increase in serum IGF-1 concentration induced by glutamine supplementation. It is also important to emphasize that glutamine may influence muscle protein balance directly. The increase in intramuscular glutamine concentration inhibits proteolysis in the muscle [40]. Apart from this effect, there is a positive relationship between the protein synthetic rate and muscle glutamine concentration up to the limit of the expansion of the intramuscular glutamine pool [41]. Wu and Thompson [42] found that elevating extracellular glutamine concentration from 1 mM to 15 mM augmented protein synthesis and decreased protein degradation in incubated chicken skeletal muscle. Nevertheless, since muscle glutamine concentration was not different among groups in the present study, it is suggested that glutamine supplementation had an indirect influence on muscle protein balance, increasing serum IGF-1 concentration. 

Despite its influence on visceral protein status, dietary glutamine supplementation did not raise glutamine concentration in plasma. This lack of effect of dietary glutamine supplementation on plasma glutamine concentration has been reported by other studies [22]. One of the possible mechanisms involved is the higher cellular uptake of glutamine when its availability in the bloodstream increases. According to Parry-Billings and Newsholme [43], the rise in plasma glutamine concentration induced by its ingestion increases muscle glutamine uptake and in consequence decreases glutamine release into circulation. Furthermore, some authors suggest that this limited effect of dietary glutamine supplementation probably reflects its utilization by enterocytes and hepatocytes [44]. 

It is well established that infectious diseases may impair growth and nutritional status. In the present study, BCG inoculation reduced body weight, lean mass, water, protein and fat content in the carcass. These results are related to the catabolic status induced by infection, which is mostly mediated by proinflammatory cytokines such as tumor necrosis factor-α [45,46]. The host responds to infection by activating its defense systems and concomitantly by mobilizing body proteins, supplying the organism with amino acids in order to meet the hypermetabolic demand induced by the infection. In growing animals, this process leads to a negative nitrogen balance and loss of body proteins [47]. It is also important to emphasize that dietary glutamine supplementation failed to reverse the detrimental effects on body composition of BCG inoculation. Nevertheless, glutamine-supplemented mice had higher fat mass when compared to control animals. This result can be attributed to the fact that glutamine carbon is utilized as a precursor for lipid synthesis in adipocytes [48]. Fatty acids produced from glutamine are incorporated into triacylglycerol in incubated adipocytes [49]. Recently, Rumberger *et al.* [50] showed that glutamine (16 mM) potentiated the glucose-dependent increase in fatty acid synthase (FAS) and glycerophosphate dehydrogenase (GPDH) mRNA in cultured primary rat adipocytes. These authors suggested that products of glutamine metabolism, such as glucosamine-6-phosphate, were important for glucose regulation of FAS and GPDH. 

## 5. Conclusion

In conclusion, the present results demonstrate that dietary glutamine supplementation had a positive influence on visceral protein status, but did not affect body composition in early-weaned mice inoculated with BCG.
